# Synthesis and complexing properties of diglycol resorcinarene podands

**DOI:** 10.1007/s10847-014-0462-y

**Published:** 2014-11-09

**Authors:** Mariusz Urbaniak, Barbara Gawdzik, Alicja Wzorek, Wiesław Kaca, Łukasz Lechowicz

**Affiliations:** 1 Institute of Chemistry, The Jan Kochanowski University, Świętokrzyska 15G, 25020 Kielce, Poland; 2 Institute of Biology, The Jan Kochanowski University, Świętokrzyska 15G, 25020 Kielce, Poland

**Keywords:** Podands, Resorcinarenes, Inclusion complex, Mass spectrometry, Antibacterial activities

## Abstract

Synthesis of new podands from resorcinarene and diethylene glycols are reported. The binding properties of these podands with alkali metal cations was studied by means of ESI–MS. The experimental results for podands with long diethylene glycol arms show the stable inclusion complexes with one or two metal cations and high affinity for sodium and potassium ions. This podands under appropriate conditions can thus form a sufficiently long cavity to accommodate more than one metal ion inside without disturbance of the axial symmetry like an ion channel. Podand with shorter arms, obtained from ethylene glycol form complexes with 1:1 stoichiometry and also readily dimers or trimers. In the presence of alkali metal cations this podand selectively binds cesium ions. The significant affinity of synthesized podands for the biologically important alkali metal ions may affect living organisms. Antibacterial activities were tested with series of Gram-positive and Gram-negative bacteria.

## Introduction

Podands which were originally simply ligands with two or more pendant polyether arms has not lost its importance today. The excellent binding properties of biological receptors are still assigned to self-assembly, molecular recognition and multivalency. Multiple-interaction ligands are useful both for cation binding and protein surface recognition, cell transfection or crystal engineering [1–5]. All these many possibilities are associated also with a wide panel of scaffolds used for the synthesis of modern podands. Good platforms for the construction of such ligands are calixarenes or cyclodextrins [6–8]. Macrocyclic oligomers with cavities, able to encapsulate guest molecules are often used alone as receptors but with the pendant polyether arms can give great results [9–14]. Resorcinarenes are similar, although the specific group of cavity-shaped supramolecular compounds and basic structural units for cavity-shaped ligands. Eight hydroxyl groups of upper rim, easily form intermolecular hydrogen bonds and, depending on the pendant arms may determine shape of cavity podands.

In this paper we reported synthesis of some new podands from resorcinarene and diethylene glycols. NMR spectroscopy and mass spectrometry were used to determine structure and composition of alkali metal complexes with podands differ in arms. Affinities and efficacies of these podands for alkali metal cations were investigated and also anti-bacterial activities were tested.

## Experimental

### Reagents and measurements

The 1H and 13C NMR spectra were measured by an NMR Bruker Avance II 600 MHz using chloroform-d as solvent. Elemental analysis was run on a Model 240 Perkin–Elmer CHN Analyzer. The ESI mass spectra were recorded on a micrOTOF-Q II (Brucker) equipped with syringe pump. The dry gas flow rate was at 4.0 l/min; the dry heater operated at 180 °C; the capillary voltage was set at 4,500 V and collision energy was at 10 eV. The sample solutions were prepared in acetonitrile–water mixture (1:1). Podands concentration was always 5 × 10^−4^ mol dm^−3^, concentrations of alkali metal chlorides were the same or larger (1 × 10^−3^ and 1.5 × 10^−3^ mol dm^−3^). Melting points were determined with a Büchi Melting Point B-540 and are uncorrected. Chromatographic separations were carried out by silica gel 60 (SiO_2_, Merck, particle size 0.040–0.063 mm, 230–240 mesh). All starting materials were purchased from Fluka and Merck and were all used as received. All solvents were of reagent grade and used without further purification.

### Synthesis of diglycol resorcinarene podands

**Scheme 1:**
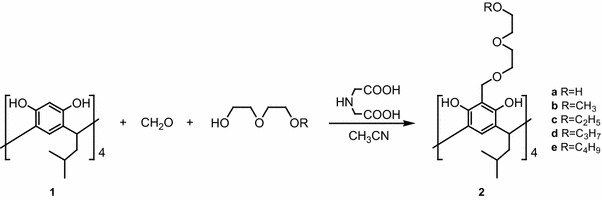
Synthesis of diglycol resorcinarene podands **2a**–**e**

The synthesis were performed by Mannich reaction under previously described conditions [15] with one slight modification (Scheme 1).

Resorcinarene **1** with formaldehyde and iminodiacetic acid were heated in a mixture of equal volume of ethylene diglycol derivatives and acetonitrile. This large excess of ethylene diglycol derivatives significantly improves yields and, in the case of diglycol ethers causes precipitation of the product from the reaction mixture. Recrystallization from acetonitrile gave pure products **2b**–**e** in 75–81 % yields. Purification of the product **2a** obtained from the reaction with diethylene glycol was more difficult. Yields after crystallization or flash chromatography on silica did not exceed 36 %. Preparation of podand **3** was carried out according to the literature [15]. 

#### Data for podand **2a**

Yield from 1 g of resorcinarene **1** (0.6 g, 36 %), R_f_ 0.17 (acetone), mp > 300 °C; δ_H_ NMR (500 MHz, CDCl_3_) 8.53 (8 H, s, ArO*H*), 7.02 (4 H, s, Ar*H*), 4.77 (8 H, s, ArC*H*_2_O), 4.38 (4 H, t, *J* 7.9 Hz, ArC*H*RAr), 3.71-3.65 (8 H, m, OCH_2_CH_2_OCH_2_CH_2_OH), 3.59-3.54 (8 H, m, OCH_2_CH_2_OCH_2_CH_2_OH), 3.50-3.46 (8 H, m, OCH_2_CH_2_OCH_2_CH_2_OH), 3.45-3.43 (8 H, m, OCH_2_CH_2_OCH_2_CH_2_OH), 2.01 (8 H, t, *J* 7.2 Hz, CHCH_2_CH(CH_3_)_2_), 1.45-1.36 (4 H, m, CH_2_CH(CH_3_)_2_), 0.94 (24 H, d, *J* 6.9 Hz, CH_2_CH(CH_3_)_2_); δ_C_ (125 MHz, CDCl_3_) 22.8, 26.0, 30.7, 42.8, 61.7, 67.5, 69.5, 72.1, 72.5, 109.5, 122.9, 124.3, 149.8; *m*/*z* HR-MS (ESI) [M+Na]^+^ calcd 1,207.63923, found 1,207.64067; Elemental Anal. calculated for C_64_H_96_O_20_ (1,185.435 g mol^−1^): C 64.84, H 8.16; found 64.98; H 7.93.

#### Data for podand **2b**

Yield from 1 g of resorcinarene **1** (1.3 g, 75 %), R_f_ 0.13 (ethyl acetate); mp > 300 °C; δ_H_ NMR (500 MHz, CDCl_3_) 8.59 (8 H, s, ArO*H*), 7.12 (4 H, s, Ar*H*), 4.79 (8 H, s, ArC*H*_2_O), 4.41 (4 H, t, *J* 6.4 Hz, ArC*H*RAr), 3.68 (8 H, dd, *J* 6.3, 2.8 Hz, OC*H*_2_CH_2_OCH_2_CH_2_OCH_3_), 3.64 (8 H, dd, *J* 5.6, 2.8 Hz, OCH_2_CH_2_OCH_2_CH_2_OCH_3_), 3.62 (8 H, dd, *J* 6.3, 3.4 Hz, OCH_2_CH_2_OC*H*_2_CH_2_OCH_3_), 3.55 (8 H, dd, *J* 5.7, 2.8 Hz, OCH_2_CH_2_OCH_2_CH_2_OCH_3_), 3.36 (12 H, s, OCH_3_), 2.03 (8 H, t, *J* 7.6 Hz, CHC*H*_2_CH(CH_3_)_2_), 1.48-1.40 (4 H, m, CH_2_C*H*(CH_3_)_2_), 0.95 (24 H, d, *J* 6.9 Hz, CH_2_CH(C*H*_3_)_2_); δ_C_ (125 MHz, CDCl_3_) 22.8, 26.0, 30.7, 42.7, 59.1, 67.8, 69.8, 69.9, 70.6, 71.9, 76.7, 109.5, 122.9, 124.2, 149.8; *m*/*z* HR-MS (ESI) [M+Na]^+^ calcd 1,263.70183, found 1,263.70080; Elemental Anal. calculated for C_68_H_104_O_20_ (1,241.541 g mol^−1^): C 65.78, H 8.44; found 65.67; H 8.35.

#### Data for podand **2c**

Yield from 1 g of resorcinarene **1** (1.4 g, 77 %), R_f_ 0.44 (ethyl acetate); mp 123.1-123.6 °C; δ_H_ NMR (400 MHz, CDCl_3_) 8.62 (8 H, s, ArOH), 7.14 (4 H, s, ArH), 4.80 (8 H, s, ArCH_2_O), 4.47-4.40 (4 H, m, ArCHRAr), 3.70-3.67 (8 H, m, OCH_2_CH_2_OCH_2_CH_2_OC_2_H_5_), 3.67-3.65 (8 H, m, OCH_2_CH_2_OCH_2_CH_2_OC_2_H_5_), 3.64-3.62 (8 H, m, OCH_2_CH_2_OCH_2_CH_2_OC_2_H_5_), 3.62-3.60 (8 H, m, OCH_2_CH_2_OCH_2_CH_2_OC_2_H_5_), 3.53 (8 H, q, *J* 7.0 Hz, OCH_2_CH_3_), 2.05 (8 H, t, *J* 7.3 Hz, CHC*H*_2_CH(CH_3_)_2_), 1.51-1.40 (4 H, m, CH_2_CH(CH_3_)_2_), 1.21 (12 H, t, *J* 7.0 Hz, OCH_2_CH_3_), 0.97 (24 H, d, *J* 6.7 Hz, CH_2_CH(C*H*_3_)_2_); δ_C_ (100 MHz, CDCl_3_) 15.1, 22.8, 26.0, 30.6, 42.7, 66.6, 67.7, 69.7, 69.8, 70.7, 76.7, 109.4, 109.9, 122.9, 124.1, 149.8; *m*/*z* HR-MS (ESI) [M+Na]^+^ calcd 1,319.76442, found 1,319.76718; Elemental Anal. calculated for C_72_H_112_O_20_ (1,297.648 g mol^−1^): C 66.64, H 8.70; found 66.59; H 8.59.

#### Data for podand **2d**

Yield from 1 g of resorcinarene **1** (1.6 g, 84 %), R_f_ 0.47 (hexane/ethyl acetate 1:6, v/v); mp 122.7-123.0 °C; δ_H_ NMR (500 MHz, CDCl_3_) 8.59 (8 H, s, ArO*H*), 7.12 (4 H, s, Ar*H*), 4.78 (8 H, s, ArC*H*_2_O), 4.41 (4 H, t, *J* 7.8 Hz, ArC*H*RAr), 3.73-3.69 (8 H, m, OCH_2_CH_2_OCH_2_CH_2_OC_3_H_7_), 3.68-3.65 (8 H, m, OCH_2_CH_2_OCH_2_CH_2_OC_3_H_7_), 3.64-3.61 (8 H, m, OCH_2_CH_2_OCH_2_CH_2_OC_3_H_7_), 3.60-3.56 (8 H, m, OCH_2_CH_2_OCH_2_CH_2_OC_3_H_7_), 3.41 (8 H, t, *J* 6.1 Hz, OCH_2_CH_2_CH_3_), 2.03 (8 H, t, *J* 7.5 Hz, CHCH_2_CH(CH_3_)_2_), 1.63-1.54 (8 H, m, OCH_2_CH_2_CH_3_), 1.49-1.40 (4 H, m, CH_2_CH(CH_3_)_2_), 0.94 (24 H, d, *J* 6.9 Hz, CH_2_CH(CH_3_)_2_), 0.88 (12 H, t, *J* 6.9 Hz, OCH_2_CH_2_CH_3_); δ_C_ (100 MHz, CDCl_3_) 10.5, 22.7, 26.2, 30.7, 42.8, 61.8, 67.7, 69.9, 70.1, 70.5, 70.7, 72.5, 73.1, 109.5, 109.9, 122.9, 124.2, 149.9; *m*/*z* HR-MS (ESI) [M+Na]^+^ calcd 1375.82702, found 1,375.82289; Elemental Anal. calculated for C_76_H_120_O_20_ (1,353.754 g mol^−1^): C 67.43, H 8.93; found 67.34; H 8.86.

#### Data for podand **2e**

Yield from 1 g of resorcinarene **1** (1.6 g, 81 %), R_f_ 0.85 (hexane/ethyl acetate 1:1, v/v); mp 123.2-124.0 °C; δ_H_ NMR (500 MHz, CDCl_3_) 8.58 (8 H, s, ArO*H*), 7.12 (4 H, s, Ar*H*), 4.78 (8 H, s, ArC*H*_2_O), 4.41 (4 H, t, *J* 7.8 Hz, ArC*H*RAr), 3.72-3.70 (8 H, m, OCH_2_CH_2_OCH_2_CH_2_OC_4_H_9_), 3.66-3.64 (8 H, m, OCH_2_CH_2_OCH_2_CH_2_OC_4_H_9_), 3.63-3.60 (8 H, m, OCH_2_CH_2_OCH_2_CH_2_OC_4_H_9_), 3.60-3.57 (8 H, m, OCH_2_CH_2_OCH_2_CH_2_OC_4_H_9_), 3.45 (8 H, t, *J* 6.6 Hz, OCH_2_CH_2_CH_2_CH_3_), 2.04 (8 H, t, *J* 7.2 Hz, CHCH_2_CH(CH_3_)_2_), 1.58-1.51 (8 H, m, OCH_2_CH_2_CH_2_CH_3_), 1.49-1.41 (4 H, m, CH_2_CH(CH_3_)_2_), 1.38-1.29 (8 H, m, OCH_2_CH_2_CH_2_CH_3_), 0.95 (24 H, d, *J* 6.3 Hz, CH_2_CH(CH_3_)_2_), 0.88 (12 H, t, *J* 7.5 Hz, OCH_2_CH_2_CH_2_CH_3_); δ_C_ (100 MHz, CDCl_3_) 13.9, 19.2, 22.8, 26.1, 30.7, 31.6, 31.7, 42.8, 61.8, 67.7, 69.8, 69.9, 70.1, 70.2, 70.5, 70.7, 71.3, 72.5, 109.5, 109.5, 122.9, 124.2, 149.9; *m*/*z* HR-MS (ESI) [M+Na]^+^ calcd 1,431.88962, found 1,431.88838; Elemental Anal. calculated for C_80_H_128_O_20_ (1,409.860 g mol^−1^): C 68.15, H 9.15; found 68.17; H 9.10.

### Antibacterial activity

The antibacterial activity of the reported podands was determined by agar plate disk diffusion method [16] against *Staphylococcus epidermidis, Bacillus subtilis, Pseudomonas aeruginosa, Escherichia coli* ISO, *Escherichia coli* B, *Proteus mirabilis* R110 (mutant Ra type of S1959), *Proteus mirabilis* R45 (Re mutant of S1959 strain), *Proteus mirabilis* S1959. The podands were dissolved in DMSO at final concentration of 34 mg ml^−1^. The cellulose disks (5 mm diameter) were sprayed 20 μl of each tested solution (680 µg/disk). Determination of the bacteria susceptibility of the new synthesized podands were performed on Mueller–Hinton agar cultivation medium (Biocorp). The following procedure was used: 100 µl of bacterial suspensions were poured onto Mueller–Hinton medium, next bacterial suspension was evenly distributed on the surface of the agar. In next step cellulose discs impregnated tested podands were laid on the Mueller–Hinton agar with bacterial cells. Petri dishes with bacterial cultures and discs with podands were incubated at 37 °C for 18 h. After this time the bacteria growth inhibition zone (in mm) were measured. Each experiments were repeated at least three times.

## Results and discussion

### Spectroscopic analyses

**Fig. 1:**
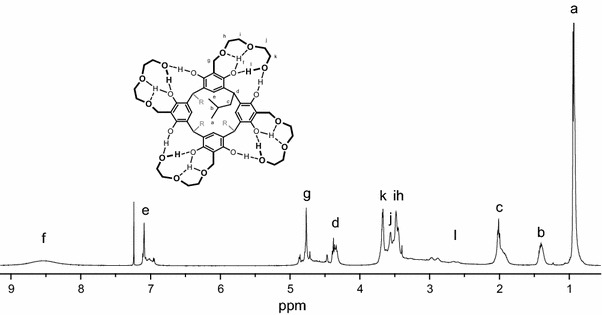
^1^H NMR spectrum of diglycol resorcinarene podands **2a** in CDCl_3_ at 25 °C

The ^1^H-NMR spectra in CDCl_3_ of podand **2a** with terminal hydroxyl groups on the pendant diethylene glycol arms is shown in Fig. 1. Compared to the previously obtained from ethylene glycol podand, the spectra show the very broad signal of the resorcinol hydroxyl protons at 8.53 ppm and signal of terminal pendant hydroxyl groups completely disappears. This indicates that diethyl glycol moiety with three oxygen atoms is appropriate length of the pendant polyether arm of resorcinarene podands to form cyclic hydrogen-bonded structure similar to crown ethers.

**Fig. 2:**
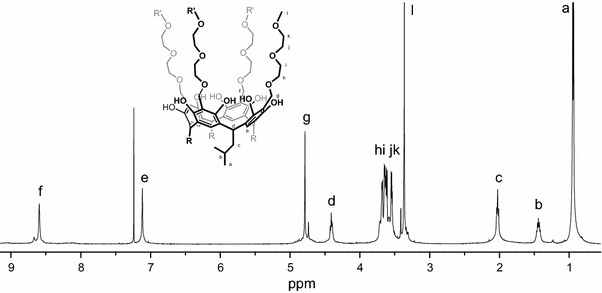
^1^H NMR spectrum of diglycol resorcinarene podands **2b** in CDCl_3_ at 25 °C

The ^1^H-NMR spectra of the podands **2b**–**e** with terminal alkoxy groups on the diethylene glycol arms are slightly different (Fig. 2). The signals are sharp as in the case of shorter podands synthesized from glycol ethers [15]. All podands obtained from resorcinarene and diglycols or their ether derivatives are thus in the crown conformation with C_4v_ symmetry. The high symmetry of the podands **2a** and **2b** is retained also in the presence of alkali cations during the titration experiments with potassium and cesium ions. This shows that the long diethylene glycol arms of podands does not interact independently with alkali metal cations by adjacent or opposite arms. All pendant diethylene glycol arms interact together and form a sufficiently long cavity to accommodate more than one metal ion inside without disturbance of the axial symmetry and a crown conformation of the resorcinarene moiety. The podands **2b**–**e** with terminal alkoxyl groups on the diethyl glycol arms under appropriate conditions can thus form an ion channel [17].

**Fig. 3:**
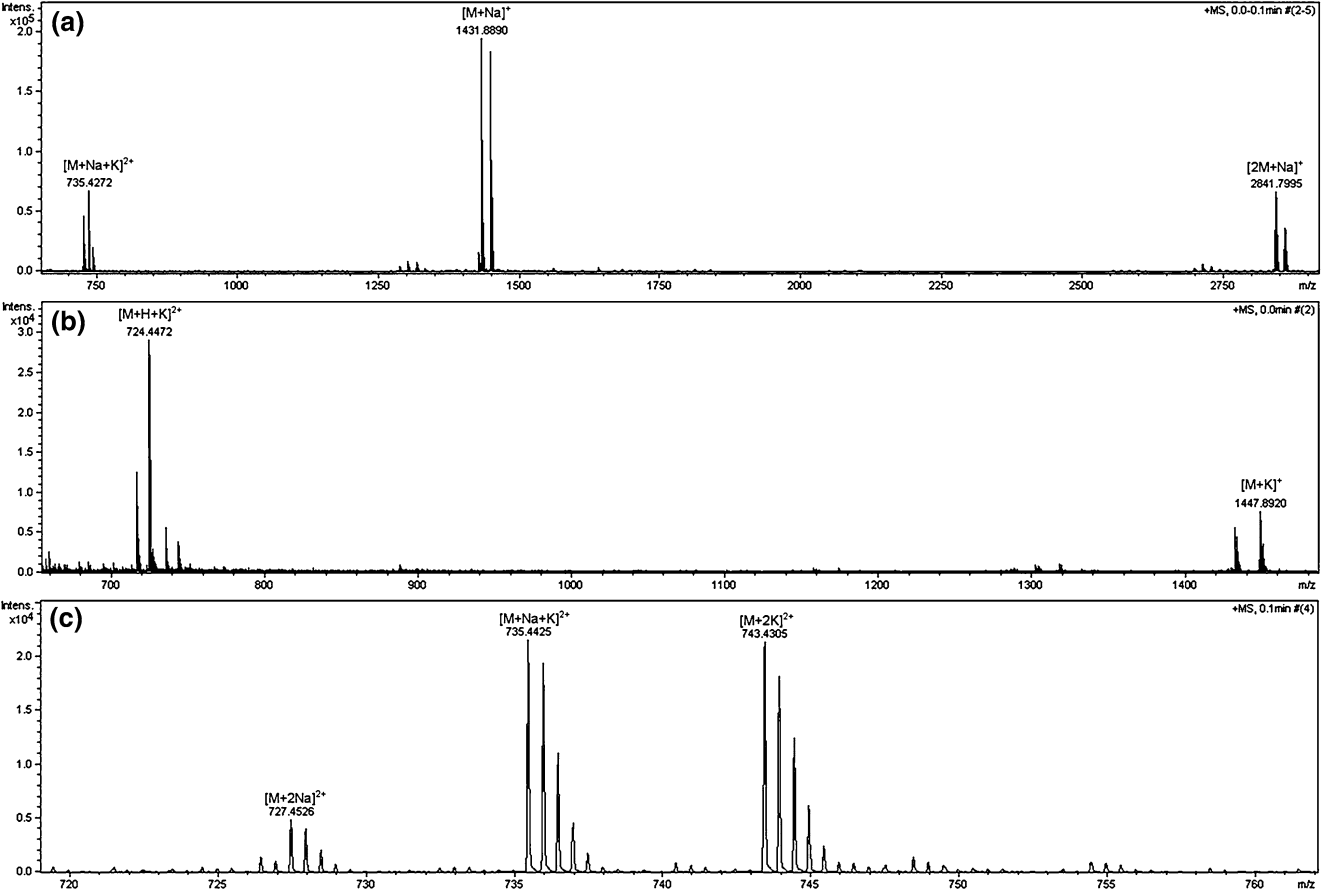
ESI–MS mass spectrum of the podand **2e**, **a** in acetonitrile–water mixture, **b** in the presence of equimolar amounts of potassium chloride, **c** in the presence of twofold excess of potassium chloride

The formation of complexes with two metal ions are confirmed by ESI–MS analysis. The spectrum of podand **2e** in acetonitrile/water (1:1, v/v) (Fig. 3a) shows mainly adducts with a single ion of metal at *m*/*z* 1,431.889 [M+Na]^+^ and 1,447.892 [M+K]^+^. The intensity of signals corresponding to adducts at *m*/*z* 735.427 [M+Na+K]^2+^ and 2,841.799 [2M+Na]^+^ are relatively low. In conditions of deficiency of cations (contamination of solvent is the only source of sodium and potassium), molecules of podand compete for sodium and potassium ions. This shows affinity of the podands to form complexes with different stoichiometry. The ESI–MS spectrum of podand **2e**, significantly changes in the presence of equimolar amounts of potassium chloride (Fig. 3b). The peaks derived from sodium and potassium adduct ions of the podand dimmers [2M+Na]^+^ and [2M+K]^+^ completely disappearing. The main peaks at *m*/*z* 716.962 [M+H+Na]^2+^ and 724.447 [M+H+K]^2+^ corresponding to doubly charged adducts with a proton and cations. The signal intensity of the adducts with one metal cation at 1,447.892 [M+K]^+^ and two metal cations at 743.430 [M+2K]^2+^ is comparable. The addition of a second equivalent of potassium chloride (Fig. 3c) shifts the equilibrium towards the adduct with two metal cations, at *m*/*z* 735.442 [M+Na+K]^2+^ and 743.430 [M+2K]^2+^ and other signals almost vanished.

**Fig. 4:**
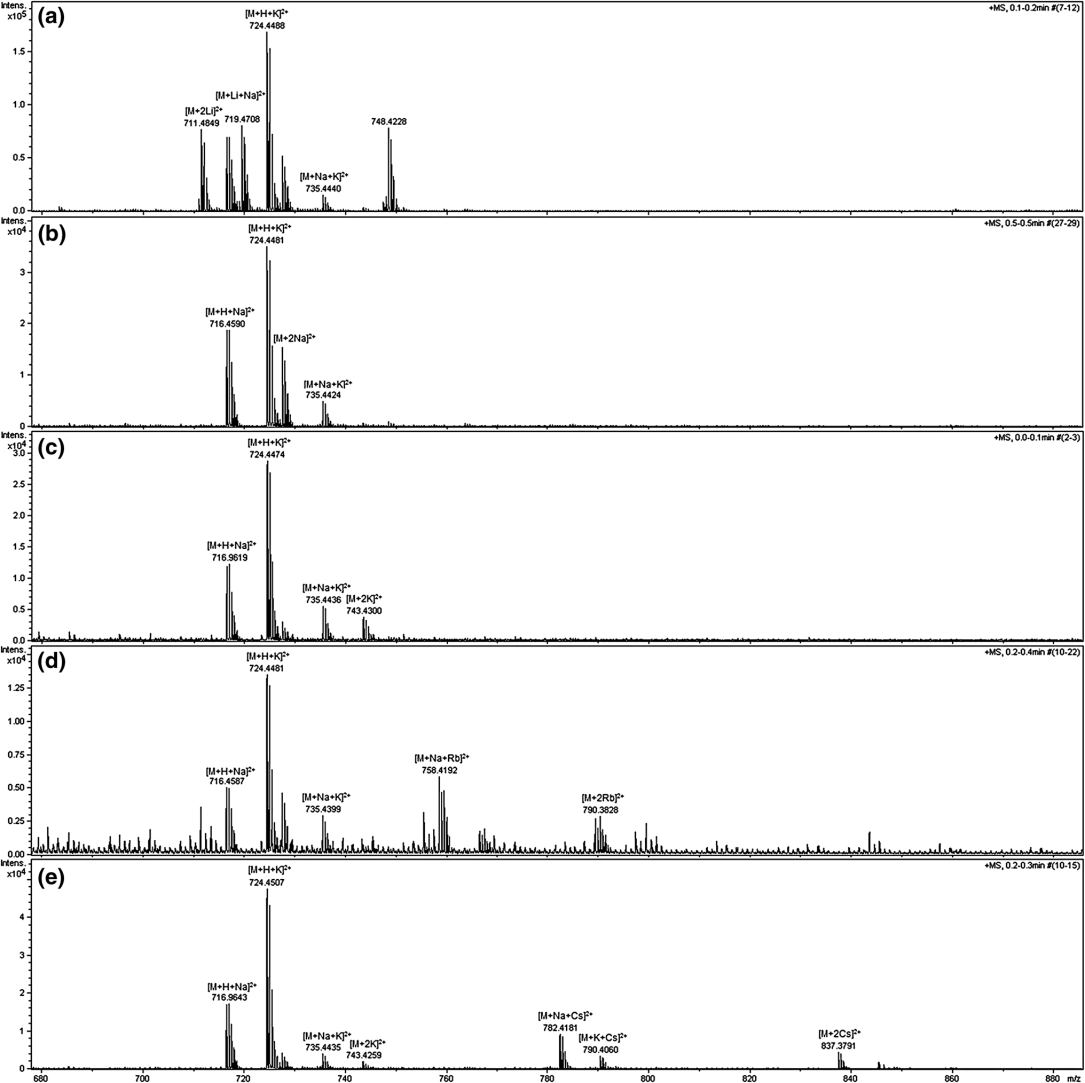
The ESI–MS spectra of **3e** and signals assigned to double charged adducts with two alkali metal ions

Similar analyses were performed also for other alkali metal cations. In each case, addition of equimolar amounts of ions resulted in the disappearance of the dimeric adduct signals and the base peak is the signal at *m*/*z* 724.448 [M+H+K]^2+^. In the doubly charged group, adducts with the same two-metal ion [M+2c]^2+^ (c = Li, Na, K, Rb and Cs) are always formed but the relative abundance decreases with increase of the ion size (Fig. 4). At this concentrations, mixed adducts, containing e.g., sodium and potassium ions also were observed. The most intense signals of adducts with one metal cation was always observed for adduct with added cation and next for the sodium adduct at *m*/*z* 1,431.889 [M+Na]^+^. To obtain some quantitative information about binding affinities of different alkali cations for podand **2e**, a competition experiment with equimolar amounts of each alkali metal cations was performed (Table 1). The main peak at *m*/*z* 1,431.889 corresponding to the sodium adduct [M+Na]^+^ of podand **2e**. The next two most intense peaks corresponding to the [M+H+K]^2+^ adduct (31 %) at *m*/*z* 724.447 and [M+2Na]^2+^ adduct (23 %) at *m*/*z* 727.453.

**Table 1:** Relative intensities of major ions in the ESI–MS spectrum obtained from equimolar mixtures of podand **2e** and alkali metal cations

Ion	M_theor_ (m/z)	M_exp_ (m/z)	Relative intensity
[M+H+Li]^2+^	708.461839	708.161758	1.33
[M+H+Na]^2+^	716.448722	716.432747	2.11
[M+H+K]^2+^	724.435691	724.446716	30.88
[M+Na+Na]^2+^	727.439694	727.453604	22.82
[M+Na+K]^2+^	735.426663	735.414286	5.74
[M+K+K]^2+^	743.413632	743.415604	1.19
[M+Li]^+^	1,415.915854	1,415.89143	1.57
[M+Na]^+^	1,431.889619	1,431.88893	100
[M+K]^+^	1,447.863557	1,447.89315	15.1
[M+Rb]^+^	1,493.811649	1,493.79692	1.45
[M+Cs]^+^	1541.805282	1541.77055	2.85

These data indicate that the podands **2b**–**e** with terminal alkoxyl groups on the diethyl glycol moieties can form with their arm a long cavity able to accommodate a two metal ions.

**Fig. 5:**
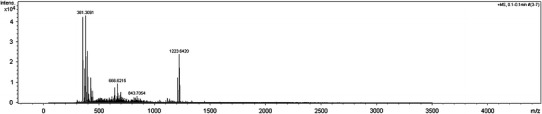
The ESI–MS spectra of **2a** in acetonitrile–water mixture

The ESI–MS spectum of the podand **2a** is completely different (Fig. 5). The most intense peaks are that for sodium [M+Na]^+^ and potassium [M+K]^+^ adduct ions at *m*/*z* 1,207.661 and 1,223.642, but dimeric or double charged adducts were not observed. When alkali metal chlorides were added to the sample solution spectra were difficult to interpret.

**Fig. 6:**
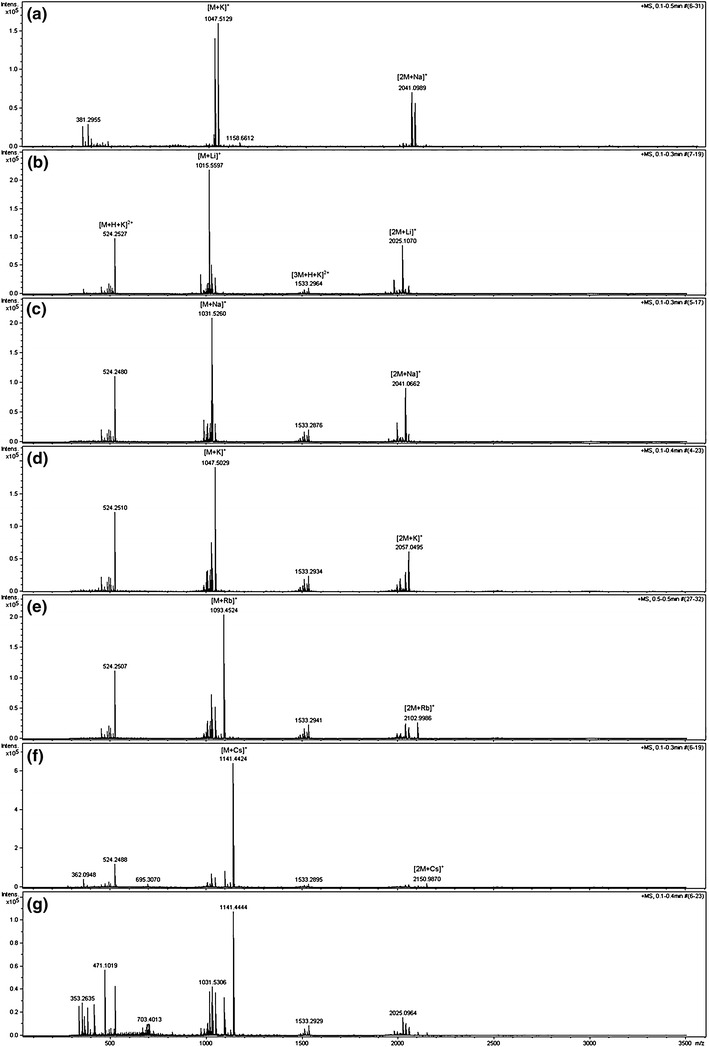
ESI–MS mass spectrum of the podand **3** **a** in acetonitrile–water mixture, in the presence of equimolar amounts of **b** LiCl, **c** NaCl, **d** KCl, **e** RbCl, **f** CsCl and **g** competitive experiment with equimolar amounts of all of these chlorides

Quite different results were obtained for podand **3** with terminal pendant hydroxyl groups and shorter arms, obtained from ethylene glycol. In the mass spectrum of pure podand **3** under the same conditions are present only signals of the monomeric at *m*/*z* 1,047.513 and dimeric at *m*/*z* 2,041.099 adducts (Fig. 6).

Addition of any alkali metal ions resulted in appearance at 524.249 strong signal of [M+H+K]^+^ adduct. This is the only signal in this doubly charged group and addition of the next equivalent of metal ion does not change the spectrum in this area. This is a significant difference compared to podands **2b**–**e**, which means that the cavity formed by the podand **3** with ethyl glycol arms, is too small for simultaneous complexation of two metal ions. The addition of metal ions also does not prevent formation of the dimers. The intensities of these peaks strongly depends on the size of the added ion and for the dimeric lithium adduct intensity of peak at *m*/*z* 2,025.107 [2M+Li]^+^ is 39 % and for dimeric adduct cesium adduct at *m*/*z* 2,150.987 [2M+Cs]^+^ only 3 %. Furthermore clear signals of doubly charged trimeric complex of podand **3** also are observed. The signal at *m*/*z* 1,533.293 [3M+H+K]^2+^ is present independently of the added metal ions while signals for the trimeric adducts with cesium and rubidium are not observed even if these ions were tested. The most intense peaks in the spectra was always observed for adduct formed with added cation [M+c]^+^ (c = Li, Na, K, Rb and Cs). In case of cesium, podand **3** selectively form the [M+Cs]^+^ adduct at *m*/*z* 1,141.442. The other adducts, are observed with very low abundance. The competitive binding experiment in the presence of equimolar alkali metal chlorides also confirmed that Cs^+^ is the preferred guest for the podand **3**.

The significant affinity of synthesized podands for the biologically important alkali metal ions inclined us to determine their effects on living organisms. The ability to form the including complexes with two ions, allows to assume that the glycol resorcinarene podands can acts as an artificial ion channels that may resulted in antibacterial activities. Resorcinarene podands antibacterial activities were tested with series of Gram-positive and Gram-negative bacteria (Table 2).

**Table 2:** The measurement of grow inhibition zone (mm) on Mueller–Hinton agar and tryptic soy agar

Bacterial strain	Mueller–Hinton agar			Tryptic soy agar	
	Podand **2a**	Podand **2e**	Podand **3**	Podand **2a**	Podand **3**
*S. aureus*	0,0,0	0,0,0	1,1,0	0,0,0	0,0,0
*S. epidermidis*	1,1,0	1,2,0	3,1,0	0,0,0	1,0,0
*B. subtilis*	0,0,0	0,0,0	0,0,0	0,0,0	0,0,0
*P. aeruginosa* PAO1	0,0,0	0,0,0	0,0,0	0,0,0	1,1,1
*E. coli* ISO	0,0,0	0,0,0	0,0,0	0,0,0	1,1,0
*E. coli* B	0,0,0	0,0,0	0,0,0	0,0,0	1,1,0
*P. mirabilis* R110	0,0,0	0,0,0	1,0,0	0,0,0	3,2,1
*P. mirabilis* R45	0,0,0	0,0,0	1,0,0	0,0,0	9,7,7
*P. mirabilis* S1959	1,0,0	2,0,0	2,0,0	0,0,0	0,0,0

The average results of three replicates

Tested podands exhibited antibacterial activity against some Gram-positive (*S. epidemidis*, *S. aureus*) as well as Gram-negative (*P. mirabilis*) strains at dose 680 µg ml^−1^. However comparison of podands **2a** and **2e** indicates that terminal groups on the pendant diether arms do not play a significant role in the interactions between podands and bacterial cells. More important, it seems, is length of the pendant arms. The podand **3**, with ethylene glycol arms exhibit somewhat enhanced antibacterial activity than the podand **2e** with longer diethylene glycol arms. This effect is even more pronounced when antibacterial tests are performed on Tryptic soy agar. When bacteria were cultivated in this medium, podand **3** have significant antibacterial properties, especially against *P. mirabilis* R45(Re). In contrary, podand **2e** does not exhibit activity at all. The differences on antibacterial activities of two R mutants—Ra and Re and smooth S 1959 strain may suggests that presence of hydrophilic polysaccharides on bacterial cells surface may prevent anti-bacterial activities of podand **3**. This result also suggested that hydrophilic surfaces like in Re mutant type (R45) are more prone towards podand **3**. It will be interesting in future to test its activities against hydrophobic cell walls of *Mycobaterium* sp. The complexation of more than one alkali ion in the podand cavity observed in ESI–MS spectra does not significantly effect on bacteria. Studies with podand dose-dependent anti-bacterial effect are needed.

## Conclusions

Summarizing, these results indicate that glycol resorcinarene podands have significant affinity for the biologically important alkali metal ions. The length of pendant arms and their terminal groups is crucial for stoichiometry of inclusion complexes. The diethylene podands with terminal alkoxyl groups and can form deep internal cavity and inclusion complexes with one or two metal cations. The competition experiment with podand **2e** and equimolar amounts of each alkali cations shows the main peak corresponding to the sodium adduct [M+Na]^+^. The next two most intense peaks corresponding to the [M+H+K]^2+^ adduct (31 %) and [M+2Na]^2+^ adduct (23 %). The podand **3** with shorter ethylene glycol arms selectively binds cesium ions and readily form dimers ant trimers. The antibacterial activity of there podands were tested with series of Gram-positive and Gram-negative bacteria. The complexation of more than one alkali ion in the podand cavity and terminal groups on the pendant arms do not play a significant role in the interactions with bacterial cells. More important, it seems, is length of the pendant arms.
